# Opinion of women about elective abortion

**DOI:** 10.4274/tjod.83723

**Published:** 2014-09-15

**Authors:** Bülent Çakmak, Fulya Zeynep Metin, Asker Zeki Özsoy, Rıza Çıtıl, Yalçın Önder, Hatice Yılmaz Doğru

**Affiliations:** 1 Gaziosmanpaşa University Faculty of Medicine, Department of Obstetrics and Gynecology, Tokat, Turkey; 2 Gaziosmanpaşa University Faculty of Medicine, Department of Public Health, Tokat, Turkey

**Keywords:** Elective abortion, Women, opinion

## Abstract

**Objective:**

The aim of this study was to investigate the opinions of women who presented to the hospital for elective abortion.

**Materials and Methods:**

This descriptive study was designed and conducted at our university hospital between March 2013-April 2013 by the method of face-to-face interviews with 500 women who presented to the hospital as patient or relatives of patients. Poll consisted of 6 questions about demographic characteristics and 14 questions evaluating the opinions and attitudes about abortion.

**Results:**

The age of the women who participated in the study was ranging between 18 and 75 years with the mean age of 31.5±11.9 years. Twenty-six women (5.2%) were illiterate, while 109 (21.8%) were university graduates. 70.8% of women stated that they were against elective abortion. Among the reasons against abortion on request were: “forbidden by the religion”-53.1% of women, “against human rights”-35.3%, and “unhealthy for the mother”-7.1% of women. About the prohibition of abortion, 82.4% of women said that “it may be performed under necessary conditions”, 9.6% “it should be completely forbidden”, and 8% stated that “it should never be forbidden”.

**Conclusion:**

A large number of respondents reported that they have negative attitude towards elective abortion, however, in case of medical necessity, abortion should be performed. During the legal arrangements done about situations that may affect the public health, such as abortion regulations, we believe it would be useful to assess the perspective of the society on this issue.

## INTRODUCTION

Abortion is the termination of pregnancy from the uterus at a woman’s own request or due to a medical requirement by a physician via several methods upon acquiring consent. In this regard, elective abortion is described by the World Health Organization (WHO) as the termination of pregnancy before the fetus has developed sufficiently to live outside the uterus^(1)^. The frequency of abortions varies, parallel with the countries and their social, cultural, and economic levels^(1,2)^. One fifth of women (during the childbearing period) in Turkey had spontaneous abortion and 22% had elective abortion according to the Turkey Demographic and Health Survey 2008 data^(3)^. In the last five years, the rate of elective abortion among the married women between the ages of 15-49 is 10% and the rate of elective abortion among the women between the 15-19 age group, which is 3%, increases with age and reaches 39% in the 45-49 age group. When analyzed according to the regions, the rate of elective abortion is the highest in İstanbul (31%) and the lowest in Southeastern Anatolia (12%). According to the education level, there is very little change to the rate of abortion among the women. However, the rate of elective abortion among women at the level of the lowest household welfare is 15% increases to 29% among women at the level of the highest welfare^(3)^.

Elective abortion was used as a family planning in the past; however, it is not used for this purpose today since there are effective family planning methods. Nevertheless, today, it is reported that one in every five women experiences one or more abortion attempts during their lifetime and there are 40 million abortion attempts every year^(4)^. Around the world, each country has its own rules and practices for elective abortion. In Turkey, the second law regulating the family planning services was re-arranged in 1983, and Article 5 of the “Population Planning Law,” Number 2827 states that: “Until the tenth week of pregnancy, the uterus can be evacuated upon request until there is no medical objection for the health of the mother”. According to this law, the elective abortions up to the 10^th^ week can be performed by competent physicians under the supervision of specialists upon the consent of the partners^(5)^. By this means, the aim is to minimize undesired pregnancies and maternal mortality rates due to the abortions carried out under improper health conditions or performed by the self-intervention of the women.

Although abortion can be performed within the frame of the legal regulations and practices, it is still among the controversial topics in terms of sociocultural, moral, philosophical, and religious aspects. The first of the two main matters of debate is “the right of fetus to live” and the other is “the right of a woman to make decisions about her own body”^(6)^. Around these arguments, maybe the women are to be emphasized and asked about their opinions. As the debate about abortion law has gained momentum, recently in Turkey in particular, an analysis of the opinions of the women via a medical disciplinary approach may be very useful and may also provide guidance on the legal practices. Due to these facts, the present study aimed to analyze the opinions of women applying to university hospitals about abortion and their attitudes towards abortion.

## MATERIALS AND METHODS

The population of this descriptive study consisted of women over 18 years of age, who applied to a University Research Hospital for any reason between March 2013 and April 2013, and agreed to participate in the study.

Based on the records, the monthly mean number of individuals who applied to a university research hospital in 2013 was 19.213; 10.960 of these individuals were women over 18 years of age. The prevalence of elective abortion from the previous studies was accepted as 22%, and the sample size calculated by Epi Info 7.0 program under a confidence interval of 97% and a deviation of d=0.04 was found to be at least 483. The study included 500 women, who were selected through random sampling among the women over 18 years of age, who applied to a hospital for any reason and agreed to participate in the study between the date ranges of the research.

A survey form consisting of 20 questions in total, “6” described the socio-demographic attributes of the participants and “14” evaluated the attitudes and opinions of the participants about elective abortion, which was prepared by the researchers for data collection as guided by the respective literature and applied to all participants through face-to-face interviews. The dependent variables of the study included the knowledge and attitudes of the women who applied to a university hospital; and the independent variables included the socio-demographic attributes of the women such as educational status, occupational status, and marital status. Prior to the study, approval was obtained from the ethics committee of the university. The data collected were analyzed using a statistical program, PAWS version 18.0. Chi-square and Fisher’s exact tests were used in the statistical analysis. A value of p<0.05 was considered as the level of statistical significance. The descriptive data of the participants were expressed in mean ± standard deviation and n (%).

## RESULTS

The mean age of the participants was 31.5±11.9 (18-75) years. Twenty-six (5.2%) women were illiterate; 141 (28.2%) were primary-school graduates; 15 (3%) were high-school students; 48 (9.6%) were high-school graduates; 161 (32.2%) were university students; and 109 (21.8%) were university graduates. The rate of participants with a profession was 25.2%, whereas 74.8% were unemployed. Regarding marital status, 55.4% were married and 44.6% were single.

The question, “Which one is more applicable to you?” regarding elective abortion was answered by 18.6% (93) as “a natural right,” 67.8% (339) as “must be performed only in health-endangering situations,” and 13.6% (68) as “absolutely not.” Seventy point eight percent (354) of the women stated that they were against elective abortion. Of the women stating that they were against elective abortion, 53.1% reported the reason for being against elective abortion as “forbidden by religion,” 35.3% as “against human rights,” and 7.1% as “unhealthy for the mother” (Table 1).

Among the questions analyzing the reasons for elective abortion from social indications, the question, “Do you approve of abortion if the married couple does not want any more children?” was answered by 18.8% as “yes”; 70.4% as “no”; and 10.8% as “undecided”. The question, “Do you approve abortion in extramarital situations?” was answered by 29.2% as “yes”; and the question “May elective abortion be performed due to financial incapacity?” was answered by 14.4% as “yes” and 73.8% as “no”.

The question, “Do you approve abortion if the married couple does not want any more children?” and the question, “Do you approve abortion in extramarital situations?” were found to receive a higher rate of “yes” by the participants over 40 years of age than the other participants (p<0.05). Additionally, the question as to whether to forbid abortion completely or not was answered as “yes” at a rate ranging between 6.8% and 13.3%, based on the age group, and the same answer applied to all age groups (p=0.202) (Table 2).

When the opinions of the participants were analyzed based on their educational levels, the highest rate of positive approach to the elective abortion was found in the group consisting of university students (39.1%; p<0.001). The frequency of “yes” as a response to completely forbid abortion based on the groups was zero (0%) of the illiterate participants, 26 (18.4) of the primary-school graduates, two (13.3%) of the high-school students, eight (16.7%) of the high-school graduates, seven (4.3%) of the university students, and five (4.6%) of the university graduates, respectively (Table 3).

Ninety-six (19.2%) of the participants answered “yes” to the question of whether they had an abortion compared to 404 (80.8%) answering “no”. When the reasons for the procedure were asked of the women who had an abortion, 26% responded with “undesired pregnancy,” 37.5% responded with “due to health problems,” 9.4% responded with “the notice from their doctors that the baby would be disabled,” and 27.1% responded with “other”. The question, “May elective abortion be performed if there are no health related issues?” was answered as “yes” by 38.5% and as “no” by 61.5% among the women who had abortion compared to the rates 27% and 73% among the women who did not have abortion, respectively (p=0.025). The question, “Do you approve of abortion if the married couple does not want any more children?” was answered as “yes” by 37.5% of the women who had an abortion compared to 14.4% among the women who did not have an abortion previously (p<0.001). Thirty-nine point six percent of the participants who had a history of abortion approved of extramarital abortion compared to 26.7% of the women who had not had an abortion (p=0.042) (Table 4).

Four hundred fifty (90%) women reported that abortion was not a contraceptive method, 2.6% reported that it might be used as a contraceptive method, and 7.4% reported that they did not have any knowledge about the matter. With regard to completely forbidding abortion by law, 48 (9.6%) of the participants answered as “it should be completely forbidden,” 412 (82.4%) answered as “it may be performed under necessary conditions,” and 40 (8%) answered as “it should never be forbidden”. The arguments of the total 354 participants who provided “no” as an answer to the question of whether to forbid elective abortion were respectively: “it should be completely forbidden” by 13%; “it may be performed under necessary conditions” by 86.4%; and “it should never be forbidden” by 0.6%.

## DISCUSSION

In the present study, 18.6% of the participants defined abortion as “a natural right” and 70.8% reported that they were against elective abortion. In addition, only 9.6% reported that abortion should be completely forbidden by law. It is understood that the majority of the participants report that they are against elective abortion; however, only 9.6% had a positive approach to the complete ban of abortion. In the study by Baykan et al. on this matter, they reported that most of the women thought that elective abortion was not appropriate when the opinions of the women regarding abortion were analyzed in moral, ethical, and social terms. Despite this, women also stated that deciding the fate of the pregnancy was a natural right of the women and the government should not intervene in elective abortion^(7)^. It is seen that abortion, which is still an ethical issue today, may be due to social causes such as rape as well as due to an undesired pregnancy resulted from not using proper and correct family planning method, and therefore service accessibility and availability and raising the awareness of women through health education in this regard are considered as the important steps for the solution of the problem^(8)^.

The study by Bilgin et al. that evaluated the opinions of university students about elective abortion reported that the students did not consider elective abortion as a family planning method, they knew its damages to the health, and although they tended to be against elective abortion, they wanted it to be a matter of free choice^(9)^. In another study in which the opinions of health college students were collected, 52.1% of the students assessed elective abortion as morally incorrect, whereas 28.7% stated that they did not share this opinion^(10)^. In the present study, 90% of the participants reported that abortion was not a contraceptive method. Additionally, 53.1% of the participants who were against abortion provided the reason for being against it as being “forbidden by religion” and only 7.1% were against as it as being “unhealthy for the mother”. It is quite interesting that the women with the history of abortion have more liberal thoughts about abortion in case of social indications such as excessive children and financial incapacity. On the other hand, in the present study, the group consisting of university students was also found to be more liberal towards abortion than the other groups with lower education levels.

It was also seen that women had an intense feeling of responsibility prior to elective abortion and the majority had feelings of guilt due to religious and moral concerns; however, 70% of these women did not change their decisions about abortion despite the feelings of guilt, fear, anger, regret, suffering, shame, and loss^(11,12,13)^. In the study by Doganer et al., half of the women stated that they would not have an abortion in case of a potential undesired pregnancy because of the thought that it would be a sin^(14)^. In the present study, it is also seen that the participants who are against abortion provided religion as the main reason for their objection (53.1%). Furthermore, 86.4% of the participants who were against abortion answered as “it may be performed only when necessary” rather than completely forbidding it.

In a study analyzing the reasons for admission to legally elective abortion among the women who applied for elective abortion, the first reason was the thought of having enough children (33.3%). This was followed by financial reasons (20.5%), having a young child (15.4%), exposure to teratogen (11.5%), own request (9%), extramarital relations (3.8%), employment (2.6%), experiencing a difficult pregnancy before (1.3%), presence of a chronic disease (1.3%), and request of the partner (1.3%), respectively^(15)^. In the present study, the first among the reasons for having an abortion among the women with a history of abortion was “health problems” (37.5%), followed by “undesired pregnancy” (26%), and “notice from the doctor that the baby would be disabled” (9.4%), respectively. It should also be kept in mind that the psychological trauma after elective abortion may also occur in the women if the health conditions of the procedural environment and pre- and post-abortion care are not sufficiently provided during the elective abortions, as well as addressing religious beliefs, such as the woman having physical health problems due to the impact of the social and cultural structure. In a study evaluating the problems and the levels of anxiety experienced by the women after elective abortion, it was reported that the most frequently reported complaint after abortion was pain (25.5%) and the second most frequently reported complaint was the sorrow due to the loss of the baby (15.4%). Moreover, it was also emphasized that the pre-procedure anxiety scores were higher than the post-procedure scores^(16)^. Psychological trauma was observed much severe among women, especially when the abortion was not legal^(17)^.

### Limitations of the Study

The results cannot be generalized to the community since the study included only the women who applied to the university hospital. It is believed that studies that employ proper sampling methods representative of the community should be conducted in order to evaluate the opinions of women in the community about elective abortion in a more correct manner.

## CONCLUSION

In conclusion, a majority of the participants has reported that they have a negative opinion about elective abortion, whereas a similar rate of the participants has stated that abortion may be performed in case of necessity. In conclusion, the authors believe that the opinions of the women should be evaluated by community-based studies as preparing legal regulations on situations that can affect the public health such as abortion, and the adverse medical outcomes due to abortion under unhealthy conditions if forbidden should also be considered.

## Figures and Tables

**Table 1:**
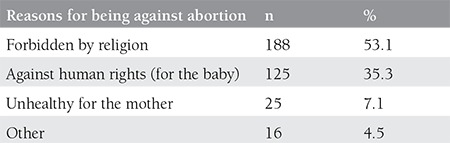
Reasons of participants for being against elective abortion

**Table 2:**
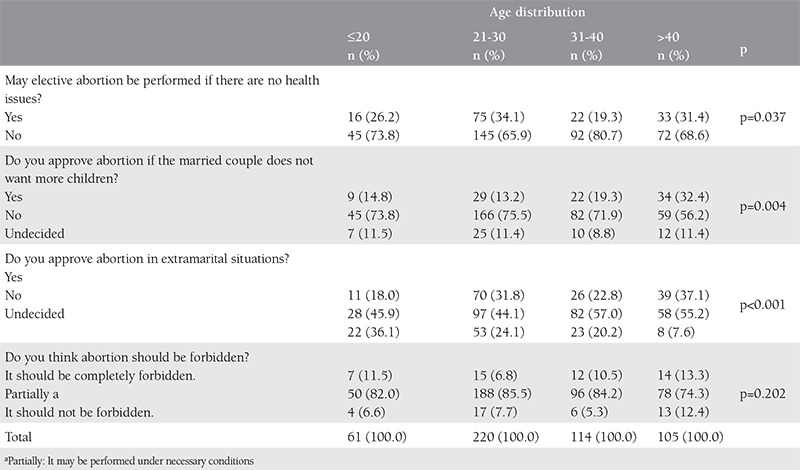
Opinions of participants about elective abortion by age distribution

**Table 3:**
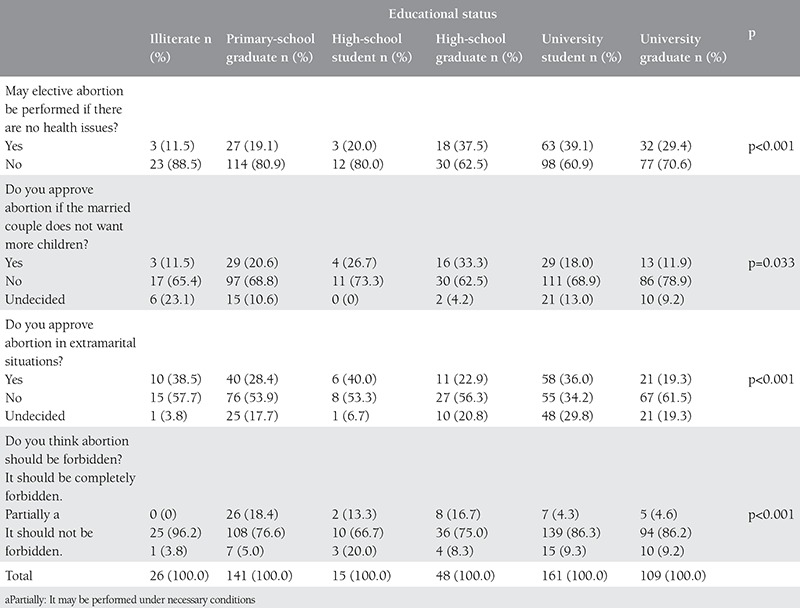
Opinions of participants about elective abortion by educational status

**Table 4:**
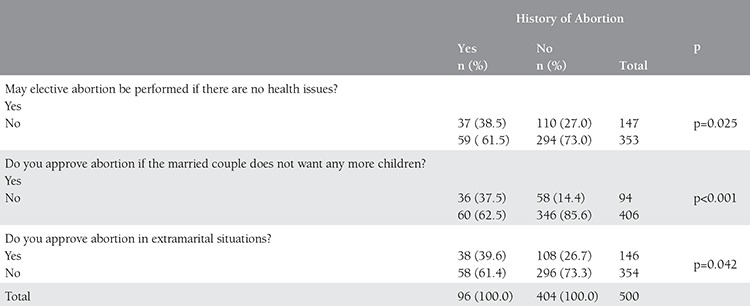
Opinions of participants about elective abortion by the history of abortion
